# Inhaled nitric oxide alleviates hyperoxia suppressed phosphatidylcholine synthesis in endotoxin-induced injury in mature rat lungs

**DOI:** 10.1186/1465-9921-7-5

**Published:** 2006-01-11

**Authors:** Xiaohui Gong, Chunbao Guo, Shibing Huang, Bo Sun

**Affiliations:** 1 Laboratory of Respiratory and Intensive Care Medicine, Children's Hospital of Fudan University, Shanghai 200032, P. R. China

## Abstract

**Background:**

We investigated efficacy of inhaled nitric oxide (NO) in modulation of metabolism of phosphatidylcholine (PC) of pulmonary surfactant and in anti-inflammatory mechanism of mature lungs with inflammatory injury.

**Methods:**

Healthy adult rats were divided into a group of lung inflammation induced by i.v. lipopolysaccharides (LPS) or a normal control (C) for 24 h, and then exposed to: room air (Air), 95% oxygen (O), NO (20 parts per million, NO), both O and NO (ONO) as subgroups, whereas [^3^H]-choline was injected i.v. for incorporation into PC of the lungs which were processed subsequently at 10 min, 4, 8, 12 and 24 h, respectively, for measurement of PC synthesis and proinflammatory cytokine production.

**Results:**

LPS-NO subgroup had the lowest level of labeled PC in total phospholipids and disaturated PC in bronchoalveolar lavage fluid and lung tissue (decreased by 46–59%), along with the lowest activity of cytidine triphosphate: phosphocholine cytidylyltransferase (-14–18%) in the lungs, compared to all other subgroups at 4 h (p < 0.01), but not at 8 and 12 h. After 24-h, all LPS-subgroups had lower labeled PC than the corresponding C-subgroups (p < 0.05). LPS-ONO had higher labeled PC in total phospholipids and disaturated PC, activity of cytidylyltransferase, and lower activity of nuclear transcription factor-κB and expression of proinflammatory cytokine mRNA, than that in the LPS-O subgroup (p < 0.05).

**Conclusion:**

In LPS-induced lung inflammation in association with hyperoxia, depressed PC synthesis and enhanced proinflammatory cytokine production may be alleviated by iNO. NO alone only transiently suppressed the PC synthesis as a result of lower activity of cytidylyltransferase.

## Background

Surfactant dysfunction and deficiency is one of the most important mechanisms in acute lung injury (ALI) and acute respiratory distress syndrome (ARDS) in adults and children [1,2]. ARDS is often encountered as a complication of various diseases or critical conditions, such as pneumonia, sepsis, pancreatitis, trauma, burn injury, cardiovascular and gastrointestinal operations, or as a part of multiple organ system failure. Pathogenesis of ALI and ARDS involves intrapulmonary neutrophil accumulation and inflammatory damage of alveolar structure, leading to impairment of lung mechanics and gas exchange, pulmonary hypertension secondary to hypoxic intrapulmonary vasoconstriction, and increased vascular-to-alveolar permeability. To prevent the development of ARDS from pulmonary infection and septic lung injury, inhaled nitric oxide (iNO) was introduced primarily as a selective pulmonary vasodilator to alter hypoxemic vasoconstriction. However, no long term efficacy was found in overall survival and ventilation time shortening despite the fact that iNO significantly reduces pulmonary hypertension and intrapulmonary shunt, thereby improving systemic oxygenation [3-7]. Attempts are made in recent years to verify therapeutic efficacy of iNO in ALI/ARDS, and it is proposed as a bridging therapy for severe hypoxemia to obviate more invasive and expensive strategies [5,8]. In contrast, iNO may be considered as a free radical and exert cytotoxicity, as it combines with superoxide at near diffusion-limited rates to produce highly reactive oxidant peroxynitrite (ONOO^-^) which may cause secondary surfactant damage. Peroxynitrite is a potent oxidizing and nitrating agent that damages a wide spectrum of biological molecules such as DNA, lipids, and proteins [9]. NO is implicated as both prooxidant and antioxidant. It can be harmful or protective, depending on the concentration and duration of exposure and on the presence of other molecules as well as homeostasis of NO metabolism and catabolism. iNO is usually used in combination with high oxygen supply in critical conditions. Its excess of oxidative radical products in hyperoxia mediate adverse effects of oxygen. Lamb et al (10) found that iNO increases 3-nitrotyrosine, a marker for ONOO^- ^formation, in bronchoalveolar lavage fluid (BALF) of ARDS patients, however, they did not find correlation between BALF nitrotyrosine levels and clinical outcome. In contrast, Honda et al [11] demonstrated that tyrosine nitration was stopped in a direct lipopolysaccharides (LPS) insult in the lungs of adult rats treated with iNO at 20 ppm.

Pulmonary surfactant consists of phospholipids, hydrophilic and hydrophobic proteins that line up in the inner alveolar surface and prevent alveolar collapse. Disaturated phosphatidylcholine (DSPC) is a major surface-active lipid component of surfactant that is produced, together with other saturated and unsaturated phospholipids, by alveolar type II epithelial cells (AEC II). Pulmonary surfactant protein A is the most abundant surfactant protein, and its nitrated form may be detected in edema fluid of patients with ALI. Whether iNO in hyperoxia would have any adverse effects in surfactant phospholipid synthesis in inflammatory injury of the mature lung remains an unanswered question. Therefore, we hypothesized that impaired PC synthesis in LPS-induced lung inflammatory injury may further be aggravated in hyperoxia, and that therapeutic dosage of iNO may not exert adverse effects in PC synthesis but anti-inflammation and anti-oxidant effects in the mature lungs. We investigated *de novo *synthesis and secretion of PC by incorporation of isotope labeled precursor, [Methyl-^3^H]-choline chloride, in adult rat lungs with inflammatory injury and subjected to a long term inhalation of NO with or without high level of oxygen, in relation to activity of cytidine triphosphorylate: phosphocholine cytidylyltransferase (CCT), a key rate-limiting enzyme in synthesis of PC, as well as activity of nuclear transcription factor-κB (NF-κB) and mRNA levels of proinflammation cytokines in the lung tissue.

## Materials and methods

### Materials

LPS (E. coli 055:B5, Catal. No. L2880), cytidine 5'-diphosphocholine (CDP-choline), cytidine 5'-triphosphate (CTP), oleic acid, phosphatidylcholine (PC), phosphocholine chloride, trichloroacetic acid were purchased from Sigma Chemical (St. Louis, MO). Activated charcoal was obtained from Fisher Chemical (Fair Lawn, NJ). The [methyl-^3^H] choline chloride (84.0 Ci/mmol), phosphoryl [methyl-^14^C] choline (55.0 mCi/mmol), cytidine 5'-diphospho [methyl-^14^C] choline (55.0 mCi/mmol) were purchased from Amersham Pharmacia Biotech (Buckinghamshire, UK). A rabbit polyclonal antibody to a synthetic peptide (DFVAHDDIPYSSA) corresponding to residues 164–176 of rat liver CCT [12] was purchased from Boster Biotech (Wuhan, China).

### Experimental animals

The Children's Hospital Scientific Committee, Fudan University, approved the experimental protocol and animal care. We followed the principles of laboratory animal care (NIH publication No. 86–23, revised 1985) and Chinese national regulation for experimental animal care. Healthy Sprague-Dawley male rats (180 to 200 g) were purchased from Bikai Ltd. (Shanghai, China). Lung inflammatory injury was induced by bolus LPS (2 mg/kg, i.v.) 24 hours prior to experiment, and these rats were referred as LPS group. A normal control group (C) received sterile NaCl injection in an identical manner. Both LPS and C groups were then randomly allocated to subgroups exposed to: room air (Air), 95% oxygen (O), room air and 20 ppm NO (NO), 95% oxygen and 20 ppm NO (ONO). NO gas was prepared in 1,000 ppm, and provided with a mass flow controller. Concentration of NO was measured with an NO/NO_2 _electrochemical analyzer (NOxBOX Plus, Bedfont Scientific Inc, Rochester, UK). The device was calibrated with 25 ppm NO and 1 ppm NO_2 _as a standard calibration gas prepared from this laboratory. No differences in body weight were found between the two groups and their sub-groups (n = 8 at 4 and 24 h, n = 6 at 8 and 12 h each), and all the animals survived the designated experimantal period.

### [^3^H]-Choline incorporation into PC

To measure surfactant PC synthesis in vivo, [Methyl-^3^H] choline chloride at 15 μCi in 15 μl diluted in 0.2 ml saline was injected intravenously for each of the animals followed by exposure of specific gas for 10 minute, 4, 8, 12 and 24 hours, respectively, and then the animals were sacrificed by overdose of intraperitoneal pentobarbital (70 mg/kg), followed by exsanguinations via abdominal aorta. The lungs were perfused with 20 ml of cooled normal saline via right ventricle to remove the residual blood. BALF was collected and pooled through 2 volumes of 6 ml (approximately 30 ml/kg body weight) of cooled saline washed in and out of the lungs, each for 4 times (totally 8 times). Its supernatant collected after removal of cell debris by centrifugation was stored at -20°C until analysis for surfactant phospholipids, proteins and the special radioactivity of phospholipid. The BAL cells were counted with a cytometer. Another 2 lavages were performed and collected fluid was deserted. The lungs were removed and the tissue was immediately homogenized in 6 ml NaCl, 10 ml methanol and 20 ml chloroform at -20°C to stop further isotope incorporation according to the method described by Jacobs et al [13].

Total phospholipids (TPL) and DSPC in BALF and lung tissue after BAL were recovered following chloroform-methanol (2:1) extraction and treatment of lipid extracts with OsO_4 _in carbon tetrachloride by aluminum phosphate column chromatography according to the methods described by Mason et al [14] and Bartlett [15]. Aliquots of TPL and DSPC in chloroform were dried under pure N_2_, then subjected to routine treatment for scintillation counting measured with a Beckman multifunctional liquid scintillation counter (LS 6500, Beckman Coulter, Toronto, ON, Canada). Special radioactivity of ^3^H was expressed as count per minute (cpm). Total proteins (TP) in BALF was measured by Lowry's method [16].

In a separate cohort, five animals of each subgroup were used for histological examination of the lungs (see below). Eight rats of each subgroup were used for CCT activity and immunoblot analysis, RT-PCR for CCT mRNA expression, and an assay for NF-κB and mRNA levels of pro-inflammatory cytokines in lung tissue according to the methods described elsewhere (see below). All these animals were not subjected to the isotope injection but identical exposure to specific gases.

### Histological and morphometric examination of lungs

The lungs from each subgroups not subjected to isotope were perfused for 30 min via the pulmonary arteries with 4% formaldehyde at a pressure of 65 cm H_2_O while both lungs were first inflated to an airway pressureof 30 cm H_2_O for 1 min, and then deflated to 10 cm H_2_O for the rest of time. Representative lung tissue blocks from all lung lobes were embedded in paraffin. Sections stained with hematoxylin-eosin were examined by light microscopy for evidence of lung injury, which was scored for edema, and neutrophil infiltration. A score scaled at 0 to 4 represents the severity: 0 for no or very minor, 1 for modest and limited, 2 for intermediate, 3 for widespread or prominent, and 4 for widespread and most prominent [17]. These works were performed in a blinded manner so that investigators were not able to identify the treatment and outcome of the animals until completion of the measurement.

### Wet-to-dry lung weight ratio (W/D)

A piece of lung tissue from right lung was cut and its wet weight was determined in an automatic electronic balance AP250D (Ohaus, Florham, NJ). The lung tissue was then put in an oven at 80°C for 48 h and weighed again to obtain its dry weight for calculation of wet-to-dry lung weight ratio [17].

### Cell fractionation

Left lung tissue was homogenated in a buffer of 145 mM NaCl, 50 mM tris (hydroxymethyl) aminomethane (Tris)-HCl (pH 7.4), 50 mM NaF, and 2.5 mM EDTA (hereafter referred to as Tris-saline). The homogenate was centrifuged at 3000 *g *for 10 min. The resulting supernatant was centrifuged at 20,000 *g *for 10 min to obtain a post mitochondrial supernatant. After an aliquot of this supernatant was stored, microsomal and cytosolic fractions were obtained by centrifugation at 100,000 *g *for 60 min. Microsomes were resuspended in a volume of Tris-saline equal to that of the cytosol. All steps were carried out at 4°C. Protein content of samples was determined [16]. Homogenates and cell fractions were stored at -70°C until cytidylyltransferase activity and protein levels were measured.

### Cytidylyltransferase activity assay

CCT activity was assayed in the forward direction by measuring the rate of incorporation of [methyl-^14^C] phosphocholine into CDP-choline. The incubation medium (0.1 ml) contained 20 mM Tris-HCl (pH 7.8), 6 mM MgCl_2_, 4 mM CTP, 1.6 mM [methyl-^14^C] phosphocholine (specific activity: 0.625 μCi/μmol), and 30 μg protein. The CCT activity of homogenate was assayed in the presence of 0.5 mM phosphatidylcholine: oleic acid (1:1 molar ratio) vesicles. These vesicles were prepared by sonication as described previously. The assays for microsomal and cytosolic were performed in the absence of 0.5 mM phosphatidylcholine: oleic acid (1:1 molar ratio) vesicles. After 30 min of incubation at 37°C, the reaction was stopped by addition of 0.1 ml of 25% (w/v) trichloracetic acid and 0.5 ml of charcoal suspension (6% charcoal in 50 mM phosphocholine). The samples were placed on ice and [methyl-^14^C] CDP-choline was isolated as described previously. The recovery (67–72%) of CDP-choline was determined in each set of assays by adding a known amount of [methyl-^14^C] CDP-choline to a complete assay mixture. All assays were corrected for background and recovery [18].

### RT-PCR for CCT mRNA

Total RNA was isolated by a single-step isolation procedure using an RNA-STAT^® ^isolation solution. A sample (2 μg) of total RNA was treated with DNase 1 to remove DNA contaminants, followed by incubation with 25 mM EDTA in a total volume of 50 μl. Samples were incubated at 37°C for 60 min, followed by 3 min at 94°C. Aliquots (2 μl) of the resulting RT-PCR products were incorporated into PCR reactions. Initially, we used these methods to detect the housekeeping gene, glyceraldehyde phosphate dehydrogenase (GAPDH), as an internal standard to assess the quality of the RNA, to correct for loading artifact of the RNA gels, and to ensure that there was no DNA contamination of the RNA preparations. The PCR was composed of 30 cycles: 94°C for 45 s, 60°C for 45 s, 72°C for 2 min, followed by 72°C for 7 min. The primer sequences for CCT-PCR were as follows:

RM CT 1 5'-AGTGCAGCGCTGTGCAGTC-3' and

RM CT 2 5'-ACAGGTCACAGCCTTGCAC-3',

These sequences correspond to +199 to +218 and +1160 to +1179, respectively, of rat CCT cDNA sequence producing a 980 bp fragment [18]. The rat-specific primers used for GAPDH resulted in a 452 bp fragment. The primer sequences for GAPDH-PCR were as follows:

RM GAPDH 1 5'-ACCACAGTCCATGCCATCAC-3' and

RM GAPDH 2 5'-TCCACCACCCTGTTGCTGTA-3'.

### Immunoblot detection of CCT

Cytosol or particulate fraction proteins (30 μg) were separated electrophoretically on 12% (wt/vol) sodium dodecyl sulfate-polyacrylamide gels and transferred to polyvinylidene difluoride membranes (Millipore, Bedford, MA). After incubation with 5% blotting grade blocker (Bio-Rad, Hercules, CA) in buffer A (24 mM Tris and 0.5 mM NaCl, pH 7.4) for 2 h at 24°C, the membrane was then incubated in buffer B (buffer A plus 3% blocker, 0.1% Tween-20, and 0.1% bovine serum albumin) with anti-rat CT serum (1:500) for 18 h at 24°C. After washing three times with 0.1% Tween-20, the membrane was incubated with sheep anti-rabbit immunoglobulin G-horseradish peroxidase (1:2000) in buffer B for 90 min, then washed twice with 0.3% Tween-20 in buffer A and twice with 0.1% Tween-20 in buffer A. The membrane was soaked in ECL reagent (Amersham, ArlingtonHeights, IL) for 2 min and exposed to X-ray film for up to 5 min [19].

### Determination of Nuclear Transcription Factor-κB (NF-κB), mRNA of proinflammatory cytokines in lung tissue

Details of preparation of nuclear protein fractions and electrophoretic mobility shift assay (EMSA) to detect DNA binding activity of NF-κB were described by Cao et al [20], and results were expressed arbitrarily as densitometric unit. Expression of mRNA of tumor necrosis factor-α (TNF-α), cytokine-induced neutrophil chemoattractant-1 (CINC-1), and interleukin-10 (IL-10) in lung tissue. The methods of RNA extraction and reverse transcription were described above in the RT-PCR for CCT mRNA. PCR was performed on 2 μl of reverse transcriptase product, containing Taq DNA polymerase, dNTP, buffer, and 0.5 μM concentrations of each gene-specific forward and reverse primers (obtained from Shenggong Biotech, Shanghai, China) in a total volume of 50 μl. Gene-specific oligonucleotide primers are listed in Table I. The methods of PCR were described by Haddad et al [21]. The level of gene expression of each transcript was normalized to that of the housekeeping gene GAPDH.

**Table 1:** Biochemical analysis of bronchoalveolar lavage fluid at 24 h of different gas exposure.

Subgroup	TPL (mg/kg)	DSPC (mg/kg)	TP (mg/kg)	DSPC/TP (μg/mg)
C-Air	7.8 ± 1.6	3.4 ± 0.4	19.2 ± 04.9	186 ± 38.9
LPS-Air	6.3 ± 1.6 *	2.7 ± 0.7 **	29.9 ± 6.0 ^#^	89 ± 12.4 ^##**^
C-ONO	7.4 ± 2.0	3.3 ± 0.5	18.0 ± 3.8	188 ± 22.9
LPS-ONO	5.4 ± 1.2	2.2 ± 0.7	38.3 ± 12.0 ^##^	63 ± 20.3 ^##*^
C-O	8.1 ± 1.2	3.3 ± 0.6	19.8 ± 6.3	176 ± 33.4
LPS-O	4.1 ± 0.8 ^##^	1.6 ± 0.4 ^##^	46.2 ± 8.8 ^##^	35.0 ± 3.8 ^##^
C-NO	7.8 ± 1.8	3.5 ± 0.7	20.2 ± 6.6	179 ± 31.0
LPS-NO	5.9 ± 1.6	2.5 ± 0.8	33.2 ± 6.8 ^##^	75 ± 14.7 ^##**^

Definition of subgroups and parameter abbreviations: C, normal control group; LPS, lipopolysaccharides-treated group; Air, room air; NO, 20 ppm nitric oxide; ONO, 95% O_2 _and 20 ppm NO; O, 95% O_2_. TPL, total phospholipids; DSPC/TPL, disaturated phosphatidylcholine to TPL ratio; TP, total proteins; DSPC/TP, DSPC to TP ratio. Values represent means ± SD (*n *= 8). Statistical analysis: ^# ^*P *< 0.05, ^## ^*P *< 0.01 versus the corresponding C subgroup; * p < 0.05, ** p < 0.01 versus LPS-O.

Malondialdehyde (MDA) was measured for lipid peroxidation with thiobarbituric acid assay [22]. Lung tissue approximately 0.2 g was homogenized with 9-fold 0.5% HTAB (0.01 mol/L sucrose, 0.01 mol/L Tris-HCL, 0.1 mmol/L EDTA-Na_2_). The mixture was then centrifuged at 4000 *g *for 15 min, 0.2 ml supernatant or standards, 0.2 ml 8.1% sodium dodecyl sulfate, 1 ml of 50% acetic acid, 3 ml of 0.8% aqueous thiobarbituric acid, and 1 ml of deionized water were added to cuvettes. After heating to 95°C for 40 minutes, the mixture was centrifuged at 3,000 *g *for 15 min, and the absorbance of the upper liquid layer was determined at 532 nm. A standard curve using tetraethyoxypropane was used to express the data in terms of MDA equivalents.

Myeloperoxidase (MPO) was determined for the relative number of PMN sequestered in the lungs. Lung tissue samples (200 mg) were homogenized in 1.8 ml of 0.5% of hexadecyltrimethylammonium bromide in 50 mM potassium phosphate buffer (pH 6.0) with detergent in an ice bath. Samples were sonicated to disrupt the granules and solubilize the MPO in the hexadecyltrimethylammonium bromide. Samples were then centrifuged at 8,000 *g *for 10 min at 4°C. Assay buffer comprised 750 μl of 1.7 mM H_2_O_2 _and 650 μl of 2.5 mM 4-aminoantipyrine with 2% phenol. An aliquot of 100 μl of supernatant of each sample was mixed into 1.4 ml of assay buffer at room temperature. Results are expressed as relative change in absorbance per minute at 460 nm. One unit of MPO was defined as causing a change of 1.0 absorbance and the data were expressed as U/g lung tissue [23].

### Statistics

Data are presented as means and standard deviation (SD). Continuous parametric data were subjected to analysis of variance(ANOVA) followed by Student-Newman-Keuls post-hoc test for between-group differences. For categorical data, a Kruskal-Wallis ANOVA was used to detect differences across the groups, followed by the Wilcoxon-Mann-Whitney test for differences between the two groups. A p value < 0.05 was regarded as statistically significant.

## Results

### [^3^H] Choline incorporation into PC

**Figure 1:**
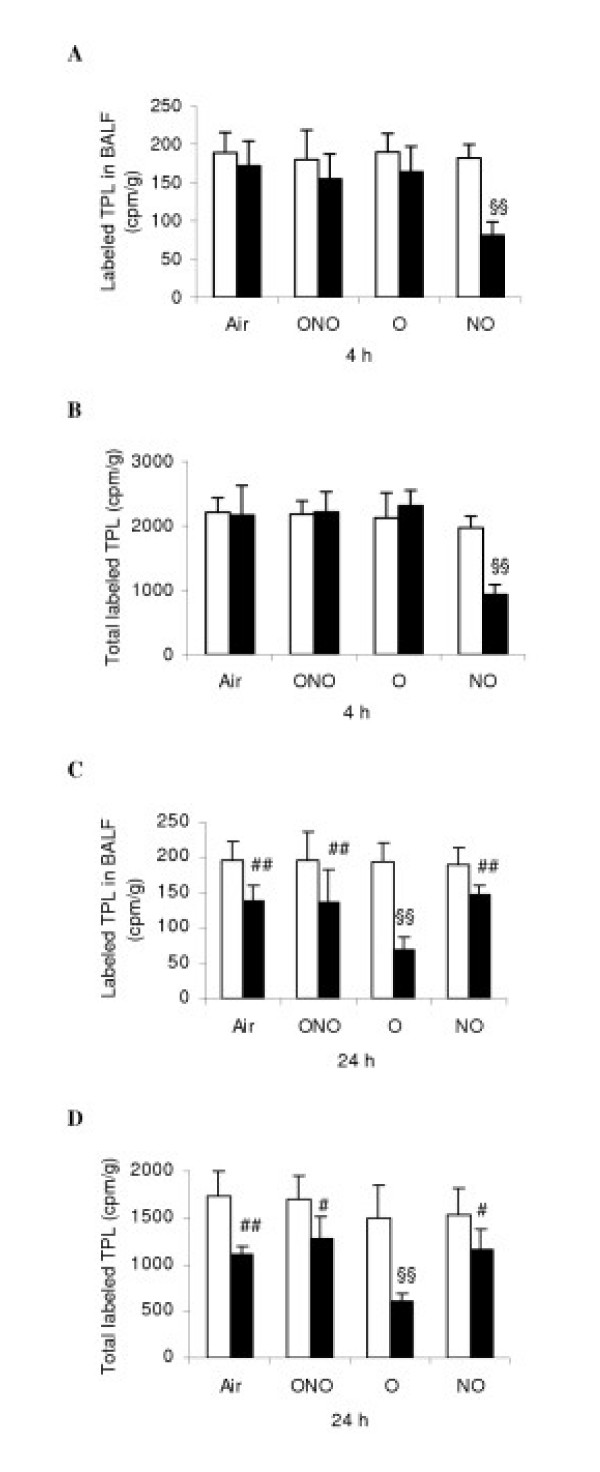
Synthesis of total phospholipids (TPL) in the mature rat lungs. The amounts of radiolabeled TPL (cpm/g body weight) recovered from bronchoalveolar lavage fluid (BALF), and in the total lungs [i.e. in both lung tissue after lavage and BALF], were determined in lipopolysaccharides (LPS)-treated (black diagrams) and non-treated control (C, white) rats. Definition of subgroups (*n *= 8 each): Air, room air; ONO, 95% oxygen and nitric oxide in 20 parts per million; O, 95% oxygen; and NO, nitric oxide 20 parts per million. Definition of each subset figures: A-B, 4 h; C-D, 24 h; A and C, [^3^H] TPL in BALF; B and D, [^3^H] TPL in the total lungs. Values are means ± SD. §§ p < 0.01 versus all other subgroups; # p < 0.05, ## p < 0.01 versus the corresponding C subgroup.

In the C-Air subgroup synthesis of PC was peaked at 4 h as reflected by specific radioactivity in [^3^H] TPL and [^3^H] DSPC, and decreased by approximately 40–50% at 24 h exposure. At 4 h, the incorporation of [^3^H] TPL in LPS-NO was significantly lower than that in all other subgroups, either in BALF or in the total lungs (BALF plus lung tissue, Fig. 1), respectively (decreased by 46~59%, p < 0.01). These differences were not significant at 8 and 12 h in which incorporation of TPL was lower by 10–20% in all the LPS-subgroups than in the corresponding C-subgroups. At 24 h, the incorporation of PC in all the LPS subgroups was significantly lower than that in the corresponding C-subgroups. A similar trend over time was seen in [^3^H] DSPC of each subgroups, which was about 20–25% in amount compared to that of the TPL (data not shown). The incorporation of [^3^H] TPL and [^3^H] DSPC in LPS-O was significantly lower than in the other LPS-subgroups (decreased by 24–47%). The secretion of PC was a ratio of [^3^H] TPL in BALF versus that in the total lungs (BALF+lung tissue). The secretion of TPL and DSPC between C- and LPS-subgroups was not significantly different at the five time points. Initial secretion rate was 10–15% at 10 min and 4 h, peaked at 12 h for [^3^H] TPL (20%) and [^3^H] DSPC (23%). At 24 h, the secretion of TPL and DSPC was decreased by 33~39% and 18~21%, respectively, compared with the peak value, respectively (data not shown).

### PC content

In general, TPL, DSPC and DSPC/TP were lower, and TP higher, in LPS-subgroups than in the corresponding C-subgroups at 4, 8, 12 and 24 h exposure. The levels of DSPC/TP, as a major marker of surfactant deficiency and/or vascular-to-alveolar permeability, in all the LPS-subgroups were consistently lower than those in the corresponding C-subgroup at different time points, and reaching statistical significance at 8, 12 (data no shown) and 24 h (p < 0.05) (Table 1). At 4 h, levels of TPL (7.6 ± 1.2 mg/kg) and DSPC (3.2 ± 0.7 mg/kg) in BALF of the LPS-NO have no significant difference with those in LPS-Air. Compared to the data at 4 h, both TPL and DSPC in BALF of the LPS-O at 24 h were significantly lower than those in LPS-Air subgroup (decreased by 35% and 41%, respectively) (Table 1). Results of biochemical analysis of TPL and DSPC in the lung tissue are shown in Table 2.

**Table 2:** Biochemical analysis of lung tissue at 24 h of different gas exposure.

Subgroup	TPL (mg/kg)	DSPC (mg/kg)	DSPC/TPL (%)
C-Air	102 ± 20.2	23.7 ± 3.9	24.1 ± 6.7
LPS-Air	96.2 ± 15.7	21.3 ± 4.2 *	22.6 ± 5.1
C-ONO	106 ± 24.3	23.7 ± 3.6	23.5 ± 6.3
LPS-ONO	101 ± 24.6	20.7 ± 4.1 *	20.8 ± 3.2
C-O	107 ± 19.9	26.6 ± 3.7	25.4 ± 5.3
LPS-O	83.2 ± 17.2	16.0 ± 2.7 ^##^	19.7 ± 3.6
C-NO	110 ± 21.4	27.4 ± 4.1	25.4 ± 4.3
LPS-NO	105 ± 17.1	24.1 ± 3.6 **	23.4 ± 4.6

For definition of subgroups and parameter abbreviations, see figure 1 legends. Values represent means ± SD (*n *= 8). Statistical analysis: ^## ^*P *< 0.01 versus the corresponding C subgroup; * p < 0.05, ** p < 0.01 versus LPS-O.

### Cytidylyltransferase activity assay

**Figure 2:**
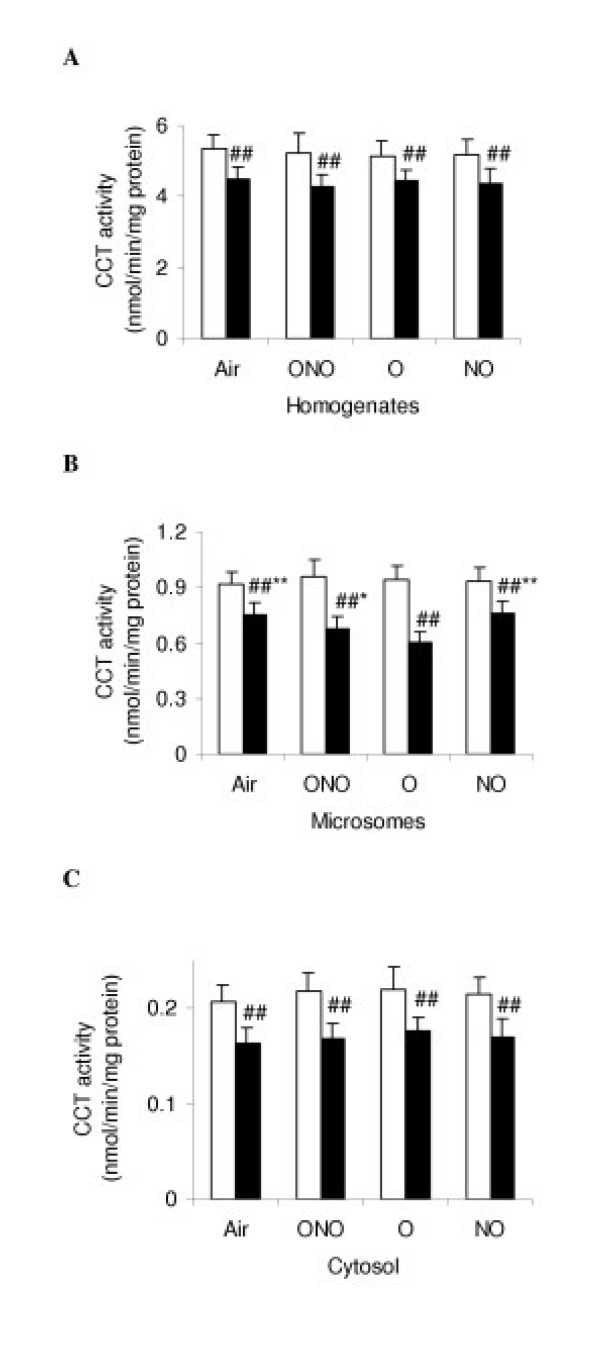
Cytidine triphosphate: phosphocholine cytidylyltransferase (CCT) activity in lung homogenates, microsomes, and cytosol at 24 h of different gas exposure in control (white diagram) and lipopolysaccharides-treated (black) rats. Definition of the subgroups see Fig. 1 legends. Definition of the subset figures: A in the presence of lipid activator; B and C, in the absence of the lipid activator. Values are means ± SD in each subgroup (*n *= 8) and expressed as nmol/min/mg protein. * p < 0.05, ** p < 0.01 versus LPS-O subgroup; ## p < 0.01, versus the corresponding C subgroups.

The CCT activity in the lung tissue homogenate was measured in the presence of lipid activator, and it represented the total amount of CCT. We found that at 4 h, there were no significant difference of CCT activity in homogenates and cytosol among all the subgroups, however, CCT activity in microsome subfraction (0.75 +0.05 nmol/min/mg protein) in LPS-NO was lower than all other subgroups (p < 0.01). At 24 h, in all the LPS-subgroups, CCT activities in homogenate, microsomal and cytosolic preparation were consistently lower than those of the corresponding C-subgroups (-20–30%, p < 0.01, Fig. 2A). CCT activity in microsome subfraction of LPS-O was significantly lower than that of the other LPS-subgroups (p < 0.05, Fig. 2B), suggesting a mechanism by which hyperoxia or iNO decreases activity of CCT is different from that by LPS.

### Measurement of CCT mRNA and protein

**Figure 3:**
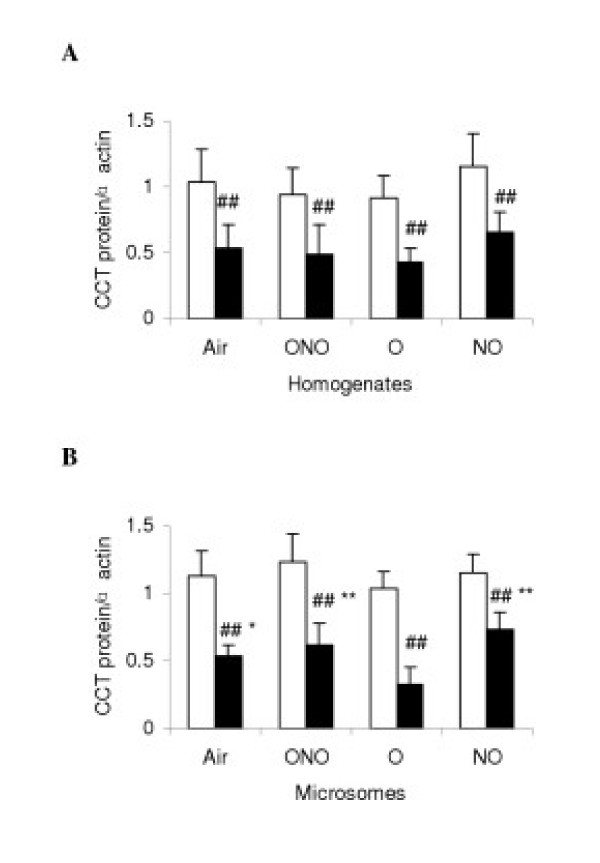
Protein contents of cytidine triphosphate: phosphocholine cytidylyltransferase (CCT) in lung homogenates (A) and microsomes (B) at 24 h of different gas exposure in control (white diagram) and lipopolysaccharides-treated (black) rats. Definition of the subgroups see Fig. 1 legends. Western blotting for CCT protein quantitated with densitometric analysis of the autoradiograms. Values are means ± SD in each subgroup (*n *= 6) and expressed as arbitrary units. * p < 0.05, ** p < 0.01 versus LPS-O subgroup; ## p < 0.01, versus the corresponding C-subgroups.

Average values of CCT mRNA/GAPDH mRNA were about 0.5–0.6 between LPS- and C-subgroups at both 4 and 24 h, with no significant significance. CCT protein/α-actin in lung homogenates and microsomes were 0.8–1.0 in C-subgroups at 4 and 24 h, with no significant differences. At 4 h, the level of this parameter for microsomal subfraction of LPS-NO was lower than all other subgroups (p < 0.05). In contrast, all LPS subgroups had significantly lower levels in the lung homogenates (0.5) than the corresponding C-subgroups (-50%, p < 0.01, Fig. 3A), however, at 24 h, in the microsomes this value was significantly lower (0.3) in LPS-O than in the LPS-NO (Fig. 3B, p < 0.01).

### Histologic and morphometric examination of the lungs

**Figure 4:**
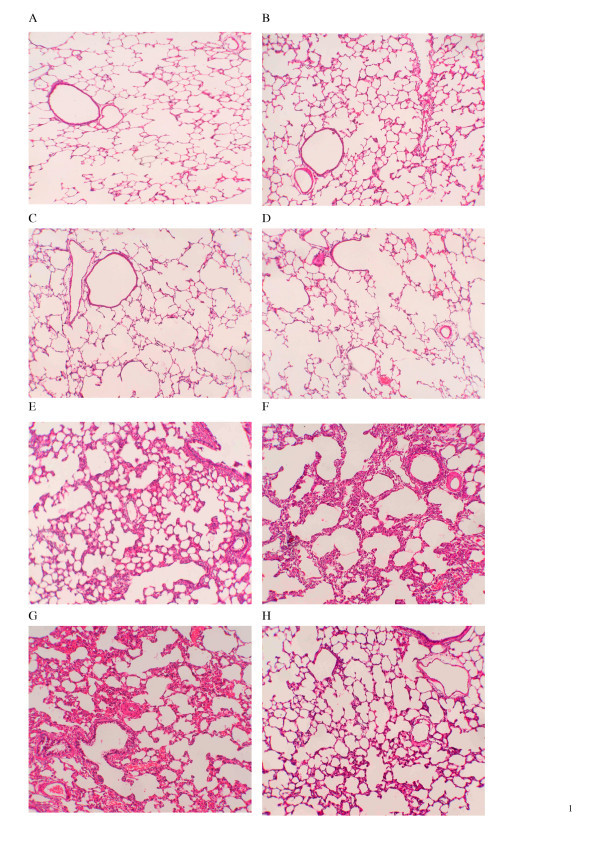
Photomicrographs of histological findings in the animal lungs at 48 h of endotoxin exposure corresponding to 24 h of different gas exposure in control (C) and lipopolysaccharides (LPS)-treated rats. Definition of the subgroups see Fig. 1 legends. All the lungs were fixed by pulmonary arterial perfusion at deflation pressure of 10 cm H_2_O for 30 min. Definition and characteristics of subset figures: A, C-Air, well aerated alveoli; B C-ONO, nearly normal alveolar structure alternative with over expanded alveoli; C, C-O, mild atelectatic alveoli; D, C-NO, nearly normal alveolar structure alternative with over expanded alveoli; E, LPS-Air, severe inflammation and edema; F LPS-ONO, severe inflammation and collapsed alternative with over expanded alveoli; G, LPS-O, very severe inflammation and collapse of alveoli; H, LPS-NO, mild collapse of alveoli. Hematoxylin and eosin staining; original magnification ×100.

As shown by lung injury score in Table 3, there were prominent edema, infiltration of neutrophils in the lungs of animals in the LPS-subgroups. At 24 h, in the LPS-O, there was deteriorated infiltration of neutrophils, but significantly alleviated infiltration of neutrophils was found in the LPS-NO (Fig. 4).

**Table 3:** Lung injury score

	4 h		24 h	
Subgroup	Edema	Infiltration	Edema	Infiltration
C-Air	0 ± 0	0.1 ± 0.2	0 ± 0	0.1 ± 0.2
LPS-Air	0.5 ± 0.4^#^	0.8 ± 0.3^#^	0.6 ± 0.2^##^	1.2 ± 0.3^##^
C-ONO	0.2 ± 0.3	0.1 ± 0.2	0.2 ± 0.3	0.2 ± 0.4
LPS-ONO	0.7 ± 0.4^#^	0.9 ± 0.4^#^	0.8 ± 0.4^#^	1.4 ± 0.2^##*^
C-O	0.1 ± 0.2	0.3 ± 0.5	0.3 ± 0.3	0.3 ± 0.4
LPS-O	0.7 ± 0.3^#^	1.0 ± 0.4^#^	1.1 ± 0.2^##^	1.9 ± 0.4^##+^
C-NO	0.1 ± 0.2	0 ± 0	0.2 ± 0.3	0.2 ± 0.3
LPS-NO	0.6 ± 0.2^#^	1.1 ± 0.4^#^	0.7 ± 0.4^#^	0.7 ± 0.3^#+^

(K-W)	p = 0.001	p < 0.001	p < 0.001	p < 0.001

*Definition of abbreviations*: (K-W), Kruskal-Wallis test of analysis of variance. Values are means ± SD (*n *= 5). For group definitions, *see *Table 1 legends. Statistical analysis: # p < 0.05, ## p < 0.01 vs the corresponding control subgroup; + p < 0.05 vs LPS-Air; * p < 0.05 vs LPS-O.

### Measurement of W/D and WCC

At 24 h, average values of W/D were higher in all the LPS subgroups (5.3–5.9) than that in the C subgroups (4.5–4.7, p < 0.01), and that for WCC were also higher in the former (10.6–26.5 × 10^6^/rat) than in the latter (2.0–2.6 × 10^6^/rat, p < 0.01), with the highest values for the LPS-O (26.5 ± 3.5 × 10^6^/rat, p < 0.05 vs. LPS-ONO).

### Measurement of NF-κB by EMSA

**Figure 5:**
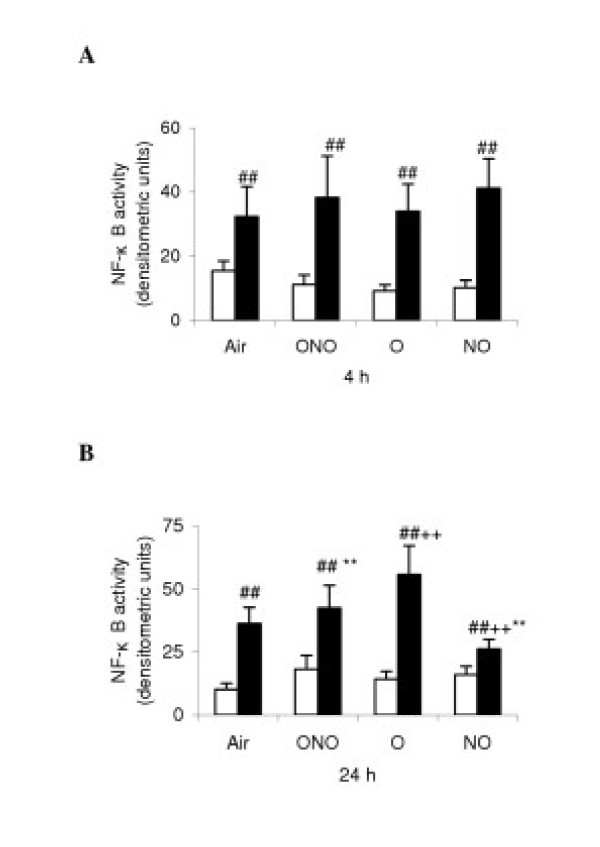
Measurement of nuclear transcription factor κB (NF-κB) in the lung tissue at 4 (A) and 24 (B) h of different gas exposure in control (white diagram) and lipopolysaccharides-treated (black) rats. Definition of the subgroups see Fig. 1 legends. NF-κB DNA-binding activity was measured with electrophoretic mobility shift assay. The values from densitometry of bands are means ± SD (*n *= 6) and expressed as arbitrary units. ++ p < 0.01 versus LPS-Air; ** p < 0.01 versus LPS-O; ## p < 0.01 versus corresponding C subgroup.

The DNA-binding activity of NF-κB in lung tissue assayed with EMSA at 4 h and 24 h revealed that the binding activity from LPS-treated rats was enhanced compared with the corresponding C-subgroups. At 24 h, the binding activity in LPS-O was higher than that of LPS-Air. On the contrary, the values in LPS-NO were lower than that of LPS-Air (Fig. 5A and 5B).

### Measurement of proinflammatory cytokine mRNA, MPO and MDA

**Figure 6:**
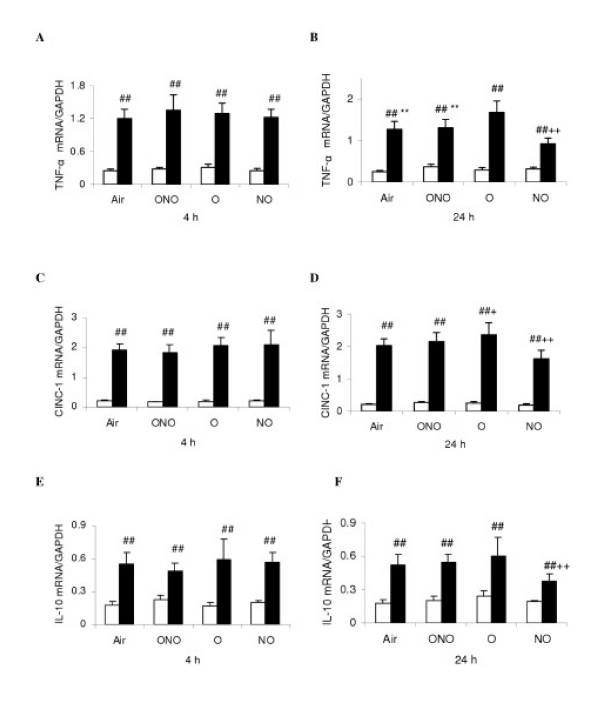
Measurement of expression of proinflammatory cytokines of the lung tissue. Definition of the subgroups see Fig. 1 legends. Definition of subset figures at 4 and 24 h, respectively, of different gas exposure in control (white diagram) and lipopolysaccharides-treated (black) rats: A-B, tumor necrosis factor alpha (TNF-α) mRNA; C-D: cytokine-induced neutrophil chemoattractant-1 (CINC-1) mRNA; E-F: interleukin-10 (IL-10) mRNA. Values are means ± SD (*n *= 6) and expressed as arbitrary units. + p < 0.05, ++ p < 0.01 versus LPS-Air; ** p < 0.01 versus LPS-O; ## p < 0.01 versus corresponding C subgroup.

**Figure 7:**
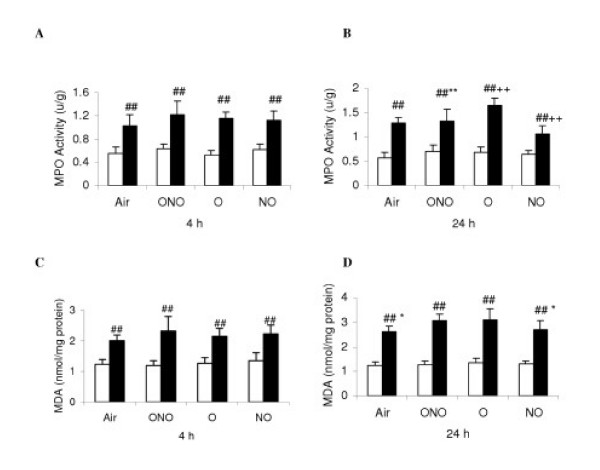
Measurement of activity of myeloperoxidase (MPO) and malondialdehyde (MDA). Definition of the subgroups see Fig. 1 legends. Definition of subset figures at 4 and 24 h, respectively, of different gas exposure in control (white diagram) and lipopolysaccharides-treated (black) rats: A-B, MPO; C-D, MDA. Values are means ± SD (*n *= 6). ++ p < 0.01 versus LPS-Air; * p < 0.05, ** p < 0.01 versus LPS-O; ## p < 0.01 versus corresponding C subgroup.

At 4 h, there was no significant difference of mRNA expression of TNF-α, CINC-1 and IL-10, and MPO and MDA levels in lung tissue among all the LPS-subgroups (Fig. 6A, C, E, and Fig 7A and 7C). After 24 h exposure, LPS-O had higher levels of TNF-α and CINC-1 mRNA, MPO and MDA than LPS-Air subgroup. LPS-NO subgroup had lower levels of TNF-α, CINC-1 and IL-10 mRNA, MPO and MDA than LPS-Air subgroup. LPS-ONO subgroup had lower levels of TNF-α mRNA and MPO than LPS-O subgroup (Fig. 6B, and Fig 7B). At all the time points, the LPS subgroups had higher levels of all the parameters than the corresponding C-subgroups.

## Discussion

In the present study, we found that inhaled NO caused a transient depression of PC synthesis in association with suppression of activity and production of CCT for PC synthesis in endotoxin-induced lung injury, however, this effect did not persist. It suggests that the therapeutic dose of iNO should not cause adverse effects in endogenous surfactant metabolism in the mature lungs with or without inflammatory injury. We also identified that iNO alleviated hyperoxia-associated suppression of PC synthesis and lung injury severity through a down-regulated NF-κB pathway for proinflammatory cytokine production. These major findings support that, as a selective pulmonary vasodilator, NO inhalation may be considered as a therapy to regulate lung inflammation, and that, at the accumulated therapeutic dose, iNO has no harm in endogenous surfactant metabolism.

Endogenous surfactant metabolism reflects function of AEC II. There are two major pathways of surfactant synthesis and utilization in the mature lung. Reuptake and reutilization of surfactant phospholipids by AEC II accounts for nearly 90% of the surfactant for daily use, and less than 10% phospholipids are synthesized through *de novo *pathway [24]. In injured lungs after birth, the pathway for reutilization of endogenous surfactant may be disturbed, and *de novo *synthesis pathway may activate again to compensate secondary surfactant deficiency and dysfunction. In this animal model, pulmonary inflammation was obvious approximately 24 h after endotoxin infusion and it caused further deterioration until 48 h, as reflected by dramatic changes in surfactant synthesis along with altered inflammatory parameters, at both 4 and 24 h of gas exposure (figures 1, 5–7), with the LPS-O subgroup being the worst one. In septic ALI. LPS triggers TNF-α releasing from alveolar macrophages and subsequently initiates an inflammatory cascade leading to diffusive lung injury [23,25]. By using low-dose intratracheal TNF-α in rats, Ulich et al showed a neutrophilic inflammatory infiltrate persisting up to 48 hours, accompanied by enhanced expression of TNF-α in AEC II [26]. The interaction among activated AM, infiltrated neutrophils, AEC I and II and vascular endothelial cells in alveolar space potentiate moderate to severe inflammation, amplifying cascade of inflammatory reaction, and inhibiting surfactant synthesis and causing surfactant dysfunction [27,28].

Miles et al [29] reported that NO generator decreases DSPC synthesis in cultured AEC II, and this result is related to decreased level of ATP intracellularly. To date, very few studies have focused on whether iNO may affect *de novo *synthesis of PC in mature lungs *in vivo *with endotoxin-induced inflammatory injury. We found that iNO transiently affected TPL and DSPC synthesis in LPS-treated rats, and had no influence on the secretion of PC. TPL and DSPC in BALF were lower in the LPS subgroups than in the corresponding C-subgroups over time, and these changes were more aggravated by hyperoxia, but less influenced by iNO as it did not affect PC synthesis in the C-subgroups. It indicates that the transient inhibition of PC synthesis did not influence PC pool size in LPS-NO treated rats as iNO was given at 20 ppm, the maximum recommended therapeutic dose for neonatal lungs. It is of interesting that iNO combined with 95% O_2 _inhibited PC synthesis only transiently in this study. Exposure of 95% O_2 _for 24 h (corresponding to 48 h of endotoxin administration) resulted in a decrease of newly synthesized [^3^H] PC as well as decreased PC pool size in LPS-treated rats. However, hyperoxia for 24 h did not affect PC synthesis in the C rats. It indicates that mature lungs with inflammatory injury is prone to oxidative injury and that iNO may alter the pathological process in this model.

Other investigations revealed that only high concentration of iNO is deleterious for mature lung, and 20 ppm of iNO should be safe in view of using this dose as the highest therapeutic concentration for ALI/ARDS as in the treatment of PPHN [30]. NO inhalation is usually used in combination with high oxygen supply in critical conditions, and may not be used with air. Previous experimental studies indicated that iNO at 100 ppm may improve survival of rats in hyperoxia [31]. However, this NO dose is irrelevant for clinical use. Issa et al [32] reported that a brief period of hyperoxia causes an oxidant stress and decreases the surface activity of alveolar surfactant in premature rabbits. In contrast, a low dose (14 ppm) of iNO decreased or prevented the O_2_-induced detrimental effects on alveolar surfactant and alleviated the oxidant stress. Mechanisms explaining the protective effects of iNO against oxidant stress my be that NO, as a weak radical acts as a scavenger of lipid radicals, terminates the cascade leading to lipid peroxidation. NO as a lipophilic agent is likely to concentrate within surfactant aggregates. In contrast, superoxide required for the formation of peroxynitrite is notably hydrophilic. Inhibition of superoxide production by neutrophils and reactions of NO inhibiting the function of ferrous iron as a transient metal are considered as alternative mechanisms against free oxygen radicals [33,34].

The biosynthesis of PC is tightly regulated within the CDP-choline pathway. A key step in this pathway is the conversion of choline phosphate to CDP-choline, which is catalyzed by the rate-limiting enzyme CCT (EC 2.7.7.15). CCT localizes primarily to the endoplasmic reticulum and nucleus, but it is also found associated with Golgi apparatus and transport vesicles. CCT activity in cells is controlled primarily by association with membrane lipids and by gene expression. In fetal lung, the cytosolic enzyme is in a predominantly inactive low-molecular-mass form, whereas in adult lung the cytosolic enzyme exists as an active high-molecular-mass multimer. Several control mechanisms for CCT have been proposed, including activation by reversible phosphorylation, membrane translocation, calcium, enhanced gene expression, and regulation of a variety of neutral and anionic lipids [35]. In our experiment, the change of CCT activity is correlated with PC synthesis. At 4 h in LPS-NO subgroup, iNO only decreased CCT activity in microsomes. Similarly, for LPS-treated rats, hyperoxia decreased CCT activity in microsomes also, and had no effect on homogenates and cytosolic subfraction. These results suggest that the decrease of CCT activity may be associated with CCT translocation from microsomes to cytosol, and forming cytosolic L form. At 24 h after the gas exposure, LPS-subgroups had a uniform decrease in CCT activity in homogenates, microsomal, and cytosolic subfractions in lung tissue compared with the C-subgroups. Mechanism of LPS induced inhibition on CCT may be different from the interventions where either iNO or hyperoxia, or both, was committed. Analysis with RT-PCR for CCT mRNA showed that iNO and/or hyperoxia did not affect CCT mRNA transcription. Furthermore, detection of immunoactive CCT protein by Western blot supports the results observed about CCT activity.

TNF-α has been implicated as a major factor for inducing ALI. Mallampalli et al [36] found that the mechanisms by which TNF-α inhibits CCT activity could be attributed to regulation of protein synthesis, changes in enzyme phosphorylation state, or induction of lipid inhibitors for the enzyme. Immunoblotting revealed that inhibition of CCT activity by TNF-α was associated with a uniform decrease in the mass of CCT in total cell lysates, cytosolic, microsomal fractions of murine AEC II. Northern blotting revealed no effects of the cytokine on steady-state levels of CCT mRNA; TNF-α did not alter *de novo *synthesis of enzyme, but it substantially accelerated turnover of CCT. We speculate that if lung inflammatory injury is alleviated, the activity of CCT should be reserved.

NO can react with O_2 _to form cytotoxic compound, nitrogen dioxide (NO_2_), and with superoxide to form a more potent molecule, peroxynitrite. Peroxynitrous acid and its decomposition products are highly reactive, promoting lipid peroxidation, nitration of tyrosine, and oxidation of DNA. Thus, one may speculate that, when superoxide is produced at high rate during inflammatory lung injury, iNO might accelerate the formation of peroxynitrite and aggravate lung damage. On the other hand, NO may act as an antioxidant to counteract the cytotoxic effects of reactive oxygen species. In vitro, NO was found to inhibit lipid peroxidation caused by oxygen radicals and to protect against cellular damage [37]. By serving as a free radical scavenger, NO may decrease lipid peroxidation *in vitro *[38,39]. In our investigation, significantly alleviated alveolar atelectasis, infiltration of neutrophils, and decreased amount of neutropils in BALF were found in the LPS-NO subgroup. NF-κB plays an important role in the initiation and progression of LPS-induced ALI. Kang et al [40] reported that iNO attenuated ALI via inhibition of NF-κB and inflammation. Kinsella et al reported a brief exposure to iNO (20 ppm for 4 h) decreased neutrophil accumulation and decreased MPO activity in ventilated ovine lungs with severe RDS [41]. We found that the exposure of 95% O_2 _for 24 h in LPS-treated rats had increased DNA binding activity of NF-κB and expression of mRNA of proinflammatory cytokines. iNO alleviated hyperoxic stress in the lungs with inflammatory injury through inhibiting activity of NF-κB and transcriptive levels of proinflammatory cytokine mRNAs in the lung tissue, and long term of therapeutic dose of iNO may not affect *de novo *synthesis of PC. Therefore we conclude that iNO should have no substantial adverse effects on metabolism and function of endogenous surfactant phospholipids through *de novo *synthesis pathway. Its anti-inflammatory effects in the mature lungs warrant further investigation.

## Declaration of competing interests

The author(s) declare that they have no competing interests.

## Authors' contributions

X. G. performed most of the experiment works and drafted the manuscript, S. H. performed isotope measurement, C. G. analyzed and intepreted NF-κB, B. S. designed experiment, supervised all experimental works and prepared manuscript. All the authors agreed to this version for submission.
